# Nonmuscle Myosin Heavy Chain ⅡA-Mediated Exosome Release via Regulation of the Rho-Associated Kinase 1/Myosin Light Chains/Actin Pathway

**DOI:** 10.3389/fphar.2020.598592

**Published:** 2020-12-08

**Authors:** Yanni Lv, Jin Chen, Jinfang Hu, Yisong Qian, Ying Kong, Longsheng Fu

**Affiliations:** ^1^ Department of Pharmacy, The First Affiliated Hospital of Nanchang University, Jiangxi, China; ^2^ Department of Neurology, The First Affiliated Hospital of Nanchang University, Jiangxi, China; ^3^ Institute of Translational Medicine, Nanchang University, Nanchang, China

**Keywords:** nonmuscle myosin heavy chain IIA, exosome release, rho-associated kinase 1/myosin light chains/actin, microglial cells, lipopolysaccharide

## Abstract

Nonmuscle myosin ⅡA, a kind of ATP-dependent molecular motor, binds actin to form the molecular motors of the cell. We found that interfering with nonmuscle myosin heavy chain (NMMHC) ⅡA could affect the exosome release from microglial cells stimulated by LPS. LPS could enhance exosome release from microglial cells by increasing exosome concentration, elevating the rate of positively labeled CD9 and CD81 proteins and protein expression. The myosin inhibitor, blebbistatin, could decrease the concentration of released exosome and reduce CD9 and CD81 protein expression on the exosome surface compared with that in the LPS group. To further determine the exact subtype of myosin Ⅱ responsible for these effects, we transfected microglial cells with siRNA for MYH9, MYH10, and MYH14. The data showed that only the transfection of siRNA-MYH9, but not MYH10 or MYH14 could decrease the released exosome concentration and particle size compared with those in the LPS group. siRNA-MYH9 would also weaken the CD9 and CD81 protein positive rate and protein expression compared with that in the LPS group by the quantification of CD9 and CD81 fluorescence intensities and by western blotting. Western blots and immunofluorescence assays indicated that NMMHC ⅡA might trigger the ROCK1/MLC/actin signaling pathway of microglial cells upon stimulation by LPS, which might be the potential mechanism of exosome release. These observations demonstrated that NMMHC ⅡA might be the potential target required for exosome release.

## Introduction

In the early stage of acute cerebral ischemia injury, the immune system is activated in response to ischemia damage. Microglia primarily act as resident immune cells of the mammalian central nervous system (CNS). Our previous research indicated that compared with neurons, the microglial cells demonstrated earlier apoptosis in response to LPS stimulation (Lv et al., 2016). A large number of inflammatory mediators are released from microglial cells, which encapsulate proteins, mRNA, miRNAs, cytokines, and other substances that are released into the circulatory system through immune defense cells (Jeppesen et al., 2019). Exosome are membrane structure vesicles with a diameter of 50 nm–1 μm. Some studies have shown that over 80% of messages are contained in the exosome. The substances in the exosomes have the characteristics of cell-cell communication (Wortzel et al., 2019), and hence can be transported to distant tissues or cells. Extensive research has been done on the function of exosomes, such as the confinement of miRNA, mRNA, and proteins in cells or tissues that secreted them. However, the process of exosome release is poorly understood. Microglia are unique innate immune cells in the central nervous system. Our previous study indicated that microglia could release micro-vesicles containing abnormally elevated expression levels of microRNAs, such as miR-146a (Lv et al., 2016) and miR-27a (Lv et al., 2018). There have been limited reports on the mechanisms of intervention in the process of exosome release by microglia cells under the stimulation of LPS. This study mainly focused on the explanation of release mechanisms of exosomes in the context of ischemia stroke diseases.

Multivesicular bodies fuse with lysosomes to deliver intraluminal vesicles for degradation. In addition, multivesicular bodies can also fuse with the plasma membrane, releasing intraluminal vesicles such as exosomes. Thus, the release mechanism might focus on the process of multivesicular bodies’ fusion. GTPases (GTP-binding proteins) (Ostrowski et al., 2010), PKM2 (pyruvate kinase type M2) (Wei et al., 2017), and TORC1 (target of rapamycin complex 1) (Zou et al., 2018) contribute to membrane trafficking and fusion into the cell. KIBRA could control exosome secretion via inhibition of the proteasomal degradation of Rab27a (Song et al., 2019). Actin-associated proteins have roles in the formation and release of exosomes (Holliday et al., 2019). As the filament slides, the head of myosin contacts actin, which provides the required forces for cytoplasmic flow, organelle movement, material transport, and mitosis, participating in cell phagocytosis and movement. Preliminary experiments indicated that myosin could interfere with the release of exosomes (Tucher et al., 2018). The specific mechanisms of intervention in the process of exosome release by microglia cells under the stimulation of LPS were investigated in this study.

## Methods

### Reagents and Materials

The microglial cells were purchased from the Beijing Beina Chuanglian Biotechnology Research Institute, China (resource number: BNCC350786) and were cultured at a density of 1 × 10^5^ cells/ml in Dulbecco’s modified Eagle’s medium (DMEM) with 10% fetal bovine serum. The medium was replaced the next day and cultured at 37°C in a humidified incubator with 5% CO_2_ and 95% room air. The following reagents and materials were used: PBS (E607008, Sangon Biotech, China), 0.45 μm filter membrane (R6BA09493; Millipore, United States), FITC Mouse IgG (400108, BioLegend, China), FITC Mouse Anti-Human CD9 (555371, Biosciences, China), FITC Mouse Anti-Human CD81 (551108, Biosciences, China), a pipettor (Research Plus, Eppendorf, Germany), a miniature refrigerated centrifuge (Microfuge 20 R, Beckman, United States), an ultra cold storage freezer (905, Thermo, United States), an ultracentrifuge (CP100MX, Hitachi, Japan), transmission electron microscopy (HT-7700, Hitachi, Japan), and a particle size analyzer (Flow NanoAnalyzer, Fuliu biotechnology company, China). The anti-CD9 (ab92726, 1:100 for WB), anti-CD81 (ab109201, 1:500 for WB), anti-nonmuscle myosin heavy chain (NMMHC) ⅡA (3,403, 1:1,000 for WB, 1:50 for IF, CST, United States), anti-phospho-NMMHC ⅡA (Ser1943) (14,611, 1:1,000 for WB, 1:50 for IF, CST, United States), anti-ROCK1 (4,035, 1:1,000 for WB, 1:100 for IF, CST, United States), anti-MLC (3,672, 1:1,000 for WB, CST, United States), anti-phospho-MLC (Thr18/Ser19) (95,777, 1:1,000 for WB, 1:800 for IF, CST, United States), and anti-β-actin (3,700, 1:1,000 for WB, 1:2,500 for IF, CST, United States) primary antibodies and Alexa Fluor^®^ 488 conjugated Donkey Anti-Goat IgG (H + L) secondary antibody (ab150129, 1:1,000, Abcam, United Kingdom) were obtained. Blebbistatin and all other chemicals used, unless otherwise stated, were obtained from Sigma Chemicals (Sigma, St. Louis, United States). NMMHC ⅡA siRNA, NMMHC ⅡB siRNA, NMMHC ⅡC siRNA, and control non-specific siRNA were synthesized by the Shanghai Biotechnology Corporation (China). siRNA transfection was performed using an ExFect Transfection Reagent (Shanghai Biotechnology Corporation, China) according to the manufacturer’s instructions.

### Exosome Extraction

Exosomes were extracted by the overspeed centrifugation method, and the extracted exosomes were analyzed by transmission electron microscopy and by particle size and nanofilm fluorescence analysis. Microglial cells were cultured at a density of 1 × 10^5^ cells/ml in DMEM medium with 10% fetal bovine serum. The medium was replaced the next day and cultured at 37°C in a humidified incubator with 5% CO_2_ and 95% room air. Microglial cells were cultured in multiple Petri dishes; a total of 20 ml of cellular supernatant was extracted. The cell suspension was centrifuged at 2,000 × g at 4°C for 30 min; the supernatant from the cell suspension was carefully moved into a new centrifuge tube and centrifuged again at 10,000 × g at 4°C for 45 min to remove the larger vesicles. The centrifugated supernatant was filtered through a 0.45 μm membrane and the filtrate was collected. The filtrate was centrifugated under overspeed centrifugation at 4°C, 100,000 × g for 70 min. The collected sediment was resuspended in 10 ml precooled 1×PBS and centrifuged again with an overspeed rotor at 4°C, 100,000 × g for 70 min. Supernatants were removed and the remaining sediment was resuspended in 50 μl pre-cooled 1×PBS; 10 μl was subjected to electron microscope observation; 10 μl was subjected to particle size detection; 30 μl was applied to fluorescence stating detection; and the remaining exosomes were frozen at −80°C.

### Cell Grouping

Protocol one: the cells were divided into three groups: control group, LPS group, and blebbistatin + LPS group. The culture medium for the culture system was DMEM medium with 10% fetal bovine serum. One mg/ml LPS (Sigma, St. Louis, MO, United States), LPS plus blebbistatin, or DMSO (Sigma, St. Louis, MO, United States) as a solvent control was added to the media. One mg/ml LPS was incubated with the microglial cells for 24 min, while 1 μM blebbistatin was added 0.5 h before the stimulation by LPS.

Protocol two: the cells were divided into eight groups: control group, NMMHC ⅡA (MYH9)-siRNA + control group, siRNA-MYH9 negative control + control group, NMMHC ⅡB (MYH10)-siRNA + control group, siRNA-MYH10 negative control + control group, NMMHC ⅡC (MYH14)-siRNA + control group, and siRNA-MYH14 negative control + control group, LPS group, NMMHC ⅡA (MYH9)-siRNA + LPS group, siRNA-MYH9 negative control + LPS group, NMMHC ⅡB (MYH10)-siRNA + LPS group, siRNA-MYH10 negative control + LPS group, NMMHC ⅡC (MYH14)-siRNA + LPS group, and siRNA-MYH14 negative control + LPS group. Microglial cells were sub-cultured in 24-well plates at a density of 3 × 10^5^ cells/ml. NMMHC ⅡA, NMMHC ⅡB, NMMHC ⅡC siRNA, and control non-specific siRNA were synthesized by the Shanghai Biotechnology Corporation (China). siRNA transfection was performed using a ExFect Transfection Reagent (Shanghai Biotechnology Corporation, China) according to the manufacturer’s instructions. Cells were transfected with siRNAs at a final concentration of 100 nM for 48 h. The siRNA sequences for NMMHC ⅡA (MYH9) were: forward, 5-GAG GCA AUG AUC ACU GACU-3 and reverse, 5-AGU CAG UGA UCA UUG CCUC-3; for siRNA NMMHC ⅡB (MYH10) were: forward, 5-CAA UGC AAU GCG GUU GCCA-3 and reverse, 5-AUA GAC GAA CGA UAC UGAC-3; for siRNA NMMHC ⅡC (MYH14) were: forward, 5-CAG UCC GCA GUA AAC UACG-3 and reverse, 5-UGA CCA GCA CGA CCG UCAG-3. To assess the siRNA silencing efficiency, total siRNA cell lysates were subjected to immunofluorescence and western blot analysis, with actin as the internal reference.

Protocol three: the cells were divided into three groups: control group, LPS group, and NMMHC ⅡA (MYH9)-siRNA + LPS group. Microglial cells were sub-cultured in 24-well plates at a density of 3 × 10^5^ cells/ml. Cells were transfected with siRNA-MYH9 at a final concentration of 100 nM for 48 h. To assess the siRNA silencing efficiency, total siRNA cell lysates were subjected to immunofluorescence and western blot analysis, with actin as the internal reference.

### Transmission Electron Microscopy of Exosomes

A volume of 10 μl of exosomes was precipitated on a copper net for 1 min; then, the floating liquid was absorbed by the filter paper. A volume of 10 μl of phosphotungstate was added to the copper network for 1 min to allow precipitation. Then, the floating liquid was absorbed by the filter paper. The samples were dried for a few minutes at room temperature. A voltage of 80 kV was selected for transmission electron microscopy and imaging.

### Exosome Particle Size Analysis

The exosomes extracted from microglia using protocol one and protocol two were subjected to particle size and concentration detection. Extracted exosomes were diluted with precooled 1 × PBS by changing the volume from 10 to 30 μl. Diluted samples were injected into a particle size analyzer. The particle size and concentration of the exosomes could be gathered after the sample was tested.

### Fluorescence Labeling and Nanoparticle Tracking Analysis of Exosomes

Exosomes are rich in proteins and have many specific marker proteins on their surfaces, such as CD9 and CD81. Four transmembrane proteins have the tetraspanin proteins CD9 and CD81, which have been shown to be involved in fusion between exosomes and cytomembranes. A volume of 30 μl of exosomes extracted from protocol two were diluted to 90 μl with precooled 1 × PBS, and then 20 μl fluorescently labeled antibodies (IgG, CD9, CD81) were added to the diluted exosomes, which were mixed and incubated at 37°C for 30 min. Then, 1 ml precooled PBS was added and samples were centrifuged at 4°C, 110,000 × g for 70 min with an overspeed rotor. The supernatant was removed carefully and precooled PBS was added, again followed by centrifugation of the samples at 4°C, 110,000 × g for 70 min with an overspeed rotor. The supernatant was carefully removed and resuspended in 50 μl precooled PBS. Diluted samples were injected into the NanoFCM instrument; after testing the sample, the protein index result was obtained. According the protocol 2, the cultured microglia were washed with PBS (pH 7.4) and fixed in ice-cold ethanol for 10 min at 4°C. Then the cells were permeabilized with 10% normal goat serum, 3% bovine serum albumin, and 0.1% TritonX-100 in PBS for 1 h, and then incubated with the primary antibody CD9 or CD81 for 48 h at 4°C. After washing, cells were incubated overnight with the secondary antibody at 4°C. The CD11b-labeled positive cells included microglia, monocytes, and macrophages. The wash step was repeated, the nuclei of all cells were then washed and incubated with 4’,6-diamidino-2-phenylindole (DAPI, ab104139, 1:500, Abcam, United States) for 15 min at 25°C. The images of morphological alterations of microglia labeled with anti-CD11b (ab8878, 1:200, Abcam, United States) were obtained under a confocal laser full scanning microscope. A histogram was constructed with the quantitative fluorescence intensities of CD9 or CD81 from a total of 45 randomly selected cells from nine independent experiments in each group of microglial cells. Nine independent experiments were analyzed.

### Immunofluorescence of Microglial Cells

According to protocol three, the cultured microglia were washed with PBS (pH 7.4) and fixed in ice-cold ethanol for 10 min at 4°C. Then, the microglia were separately permeabilized with 10% normal goat serum, 3% bovine serum albumin, and 0.1% TritonX-100 in PBS for 1 h, and then incubated with the primary antibody CD11b for 48 h at 4°C. The CD11b-labeled positive cells included microglia, monocytes, and macrophages. After washing, microglia were incubated overnight with the secondary antibody at 4°C. The wash step was repeated. The nuclei of all cells were then washed and incubated with 4,6-diamidino-2-phenylindole (DAPI, 4,083, 1:500, Millipore, United States) for 15 min at room temperature. Microglia were labeled with NMMHC ⅡA, ROCK1, *p*-MLC, MLC, and actin antibodies, which were estimated as previously described under confocal images.

### Western Blot Analysis

According to protocols one, two, and three, each microglia group was subjected to western blot analysis to detect protein expression. The microglial lysates were centrifugated at 12,000 × g at 4°C for 15 min. The supernatant was collected, and protein concentrations were determined by the bicinchoninic acid BCA assay. Samples obtained from exosome extraction protocols one, two, or, three and containing 40 μg proteins were analyzed by 12% SDS-polyacrylamide gels. The protein bands were transferred onto PVDF membranes in a Tris-glycine transfer buffer. The membranes were blocked with 5% nonfat dry milk for 2 h at room temperature and then incubated with primary antibodies: anti-CD9, anti-CD81, anti-NMMHC ⅡA, anti-ROCK1, anti-*p*-MLC, anti-MLC, or anti-actin. The incubation membrane was washed in TBST and then incubated in the appropriate AP-conjugated secondary antibody (diluted 1:2,000 in secondary antibody dilution buffer) for 1 h at 37°C. The bands were visualized with enhanced chemiluminescence and the images were captured using a bio-Image Analysis System (Bio-Rad, Hercules, CA, United States).

### Statistical Analysis

All values were expressed as mean ± standard deviation (SD). The differences between the groups were compared using a one-way ANOVA followed by Tukey’s post hoc test. An unpaired Student’s t test was used to compare the data between the two groups. The levels of significance were set to *p* < 0.05 and *p* < 0.01.

## Results

### Detection of Exosome Morphology, Diameter, Concentration, and Protein Label

**FIGURE 1:**
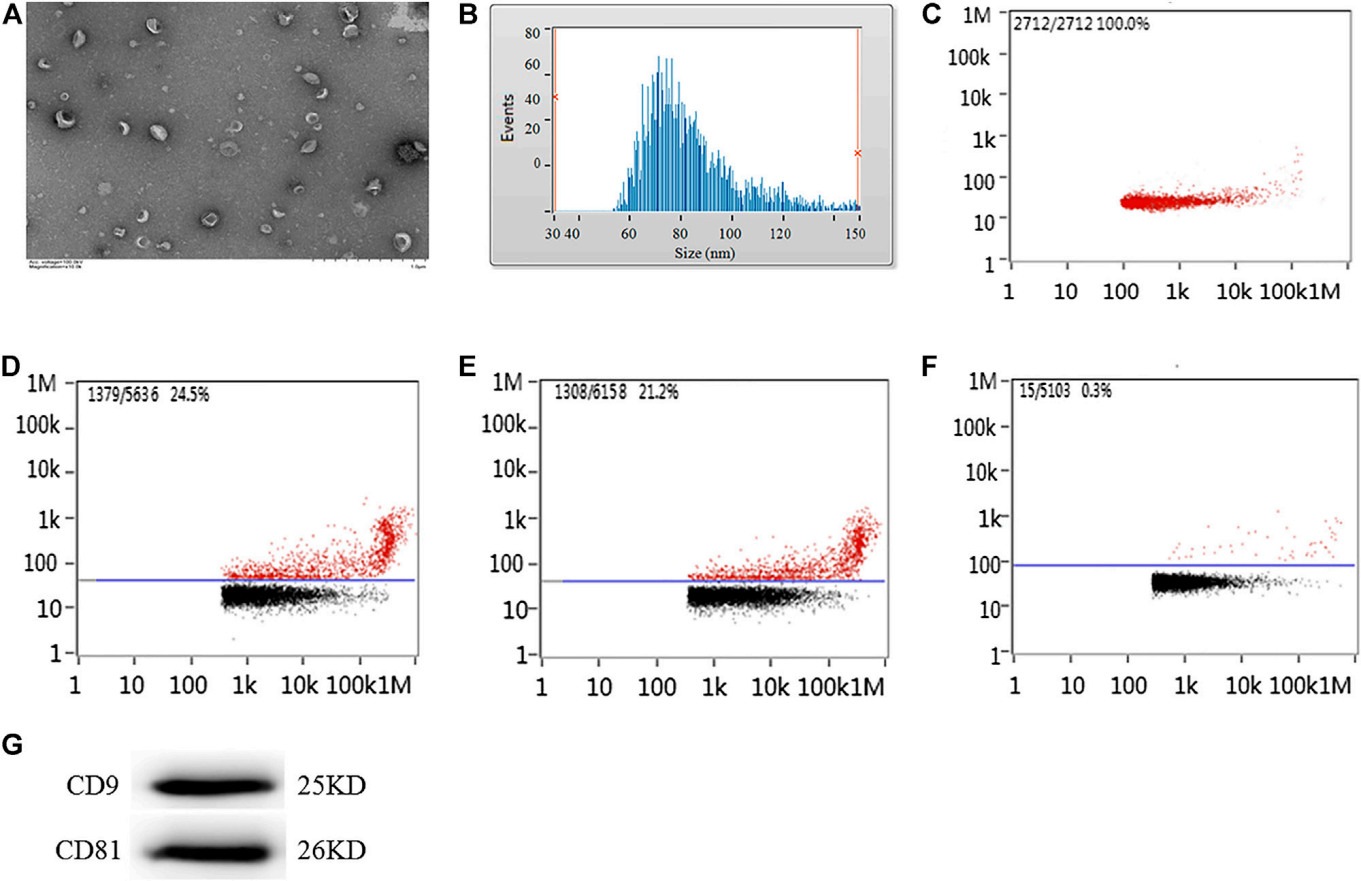
Morphology, diameter, concentration, and protein labeled positive rate detection for exosomes extracted from the supernatant of microglial cells cultured for 24 h. **(A)** Transmission electron microscopy analysis for morphology of exosome. Scale bars = 500 nm. Nanoparticle tracking analysis for **(B)** diameter of exosome or **(C)** concentration of exosome. Flow cytometry analysis for **(D)** CD9-labeled positive rate, **(E)** CD81-labeled positive rate, or **(F)** control FITC-IgG labeled positive rate. **(G)** The protein expression of CD9 and CD81 exosome marker proteins were examined by western blot. Western blots were quantified. Nine independent experiments were analyzed.

The exosomes extracted from the supernatant of microglial cells were detected under transmission electron microscopy. Transmission electron microscopy images demonstrated that micro-vesicles conformed to the morphological characteristics of the exosomes (Figure 1A). The diameters of the exosome mostly ranged between 50 and 300 nm. The average diameter was 84.28 ± 7.78 nm (Figure 1B) with a concentration of 8.89 ± 1.07×1,010 particles/ml (Figure 1C). Nanoparticle tracking analysis demonstrated that the CD9-labeled exosome surface protein positive rate was 23.98 ± 2.41%, the CD81-labeled positive rate was 22.13 ± 1.78%, and the homologous antibody control FITC-IgG-labeled positive rate was 0.21 ± 0.07% (Figures 1E, F), while the CD9 and CD81 proteins were also expressed as the marker proteins in the exosome (Figure 1G).

### Myosin Inhibitor, Blebbistatin Inhibits Exosome Release From Microglial Cells Under LPS Stimulation

**FIGURE 2:**
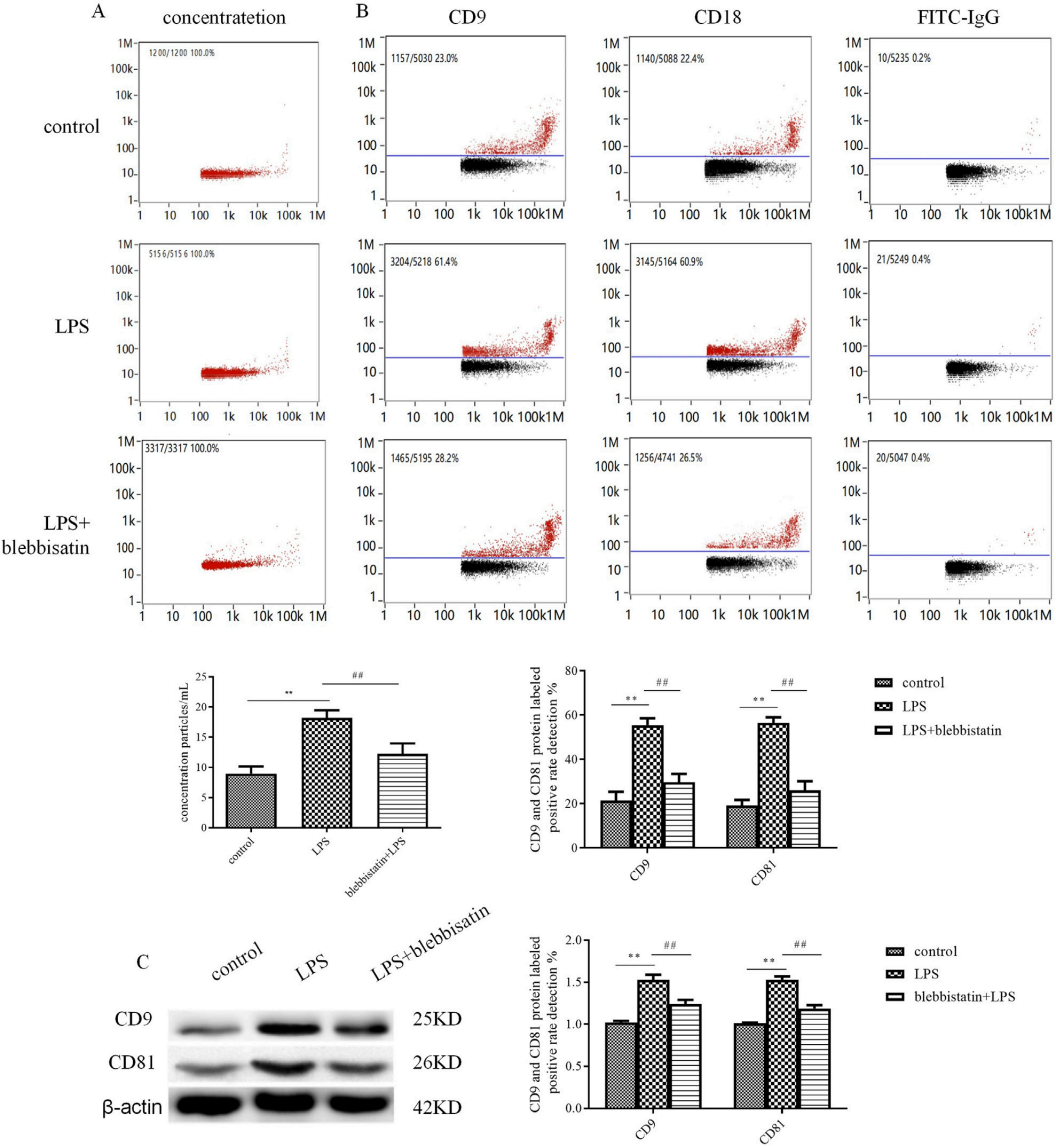
The myosin inhibitor, blebbistatin, inhibited exosome release from microglial cells under the stimulation of LPS. The extracted exosomes were divided into those extracted from control microglial cells, microglial cells incubated with 1 mg/mL LPS for 24 h, and microglial cells added with 1 μM blebbistatin 0.5 h before the 24 h stimulation of LPS. **(A)** Nanoparticle tracking analysis for the concentration of exosomes. **(B)** Flow cytometry analysis for CD9-labeled positive rate, CD81-labeled positive rate, or control FITC-IgG-labeled positive rate. **(C)** The protein expression levels of CD9 and CD81 exosome marker proteins were examined by Western blot. Western blots were quantified. Nine independent experiments were analyzed. A histogram depicts the quantitative representation of CD9 and CD81 protein expression for each group. The data were averages ±SD, n = 9. **p < 0.01 vs. control microglial cells; ^##^ *p* < 0.01 vs. microglial cells stimulated with LPS.

Compared with the LPS group, exosomes extracted from microglial cells had a similar morphology and diameter as the control group but had an increased exosome concentration and a CD9 and CD81 antibody-labeled positive rate. The exosome concentration in the LPS stimulation group was 18.30 ± 1.43×1,010 particles/ml (*p* < 0.01), while it was increased compared with that in the control group (8.56 ± 1.16 × 1,010 particles/ml) (Figure 2A). Meanwhile, the CD9 and CD81-labeled exosome surface protein expression positive rates were 57.99 ± 3.81% and 58.73 ± 3.68% (*p* < 0.01) under the stimulation of LPS, respectively; these were obviously increased compared with that of the control group (24.34 ± 3.94% and 19.09 ± 2.61%) (Figure 2B). The results of western blotting were consistent with those of CD9 and CD81-labeled protein expression. The CD9 and CD81 protein expression levels were upregulated (CD9: 1.53 ± 0.06, *p* < 0.01; CD81: 1.53 ± 0.04, *p* < 0.01) via the stimulation of LPS compared with those in the control group (CD9: 1.02 ± 0.02; CD81: 1.01 ± 0.01) (Figure 2C). After administration of the muscle myosin Ⅱ inhibitor, blebbistatin, the exosome concentration and antibody-labeled positive rate decreased. Blebbistatin decreased the concentration of exosome to 12.40 ± 1.71 × 1,010 particles/ml (*p* < 0.01), and decreased the CD9 and CD81-positive rate to 28.42 ± 4.20% and 26.02 ± 4.06% (*p* < 0.01), the CD9 and CD81 protein expression levels were also decreased (CD9: 1.24 ± 0.05, *p* < 0.01; CD81: 1.23 ± 0.04, *p* < 0.01), compared with that in the LPS group. Blebbistatin is antagonistic to the decline in release of exosomes induced by LPS.

### Knockdown of NMMHC ⅡA, but Not NMMHC ⅡB or NMMHC ⅡC Could Attenuate the Elevated Release of Exosomes Induced by LPS

**FIGURE 3:**
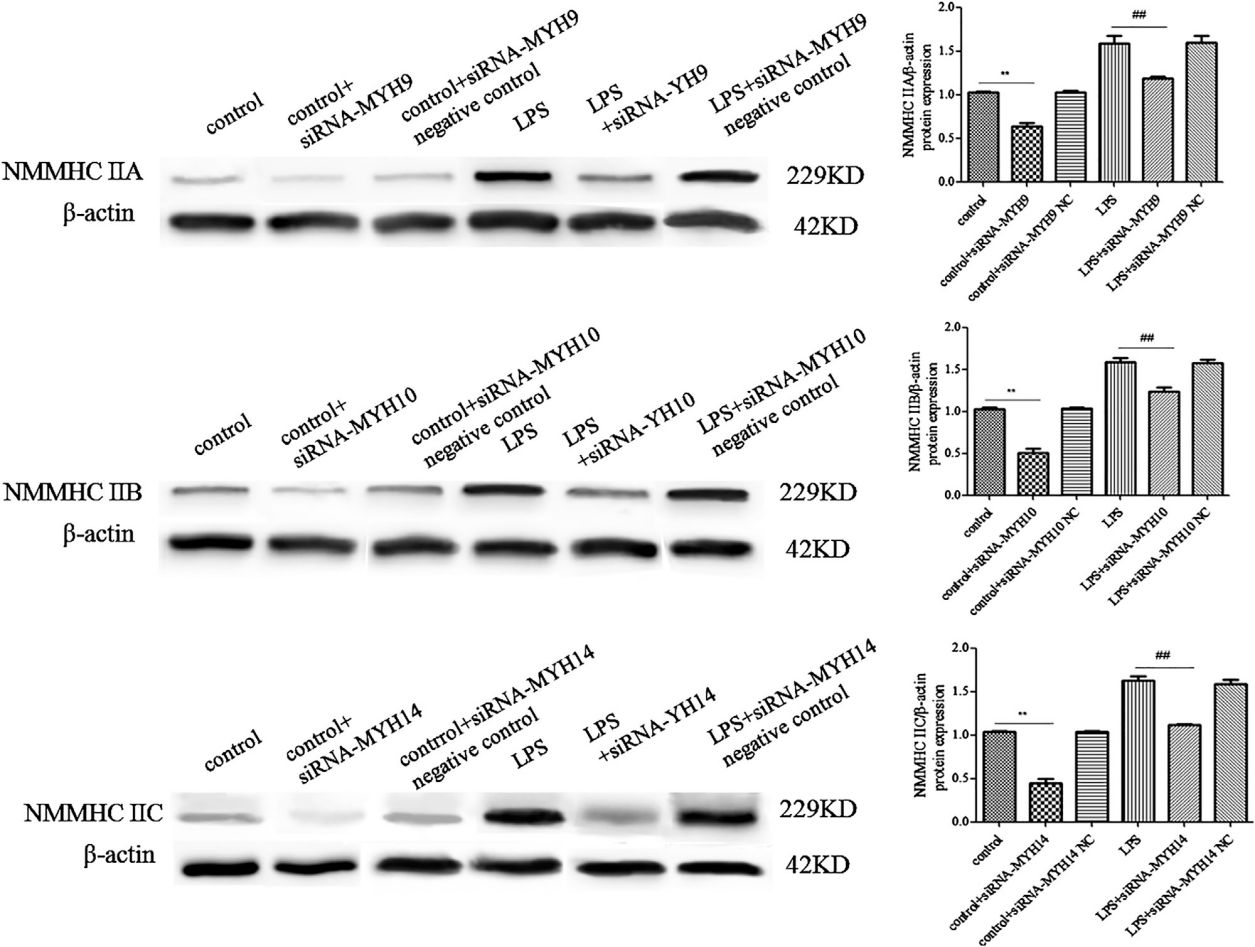
Detection of NMMHC ⅡA, NMMHC ⅡB, and NMMHC ⅡC in exosomes extracted from microglial cells. For transfection, microglial cells were sub-cultured in 24-well plates at a density of 3 × 10^5^ cells/ml. Cells were transfected with siRNA-MYH9, siRNA-MYH10, or siRNA-MYH14 at a final concentration of 100 nM for 48 h. The extracted exosomes were grouped based on whether they were extracted from control microglial cells, microglial cells transfected with siRNA, microglial cells transfected with siRNA negative control, microglial cells stimulated by 1 mg/ml LPS for 24 h, microglial cells transfected with siRNA followed by 24 h LPS stimulation, or microglial cells transfected with siRNA negative control followed by 24 h LPS stimulation. The NMMHC ⅡA, NMMHC ⅡB, and NMMHC ⅡC protein expression levels for each group of exosomes were examined by western blotting. Western blots were quantified. Nine independent experiments were analyzed. A histogram depicted the quantitative representations of the protein expression levels of CD9 and CD81 for each group. The data were averages with SD, *n* = 9. ^**^ *p* < 0.01 vs. control microglial cells; ^##^ *p* < 0.01 vs. microglial cells stimulated with LPS.

Whether in the control or LPS-stimulated microglial cells, microglial cells transfected with siRNA of NMMHC ⅡA (siRNA-MYH9), NMMHC ⅡB (siRNA-MYH10), or NMMHC ⅡC (siRNA-MYH14) could effectively interfere with the corresponding protein expression, while siRNA negative control had no effect on protein expression (Figure 3). There was not much change in the morphology and diameter of exosomes in each group, while the exosome concentration and the CD9 and CD81-labeled positive rates were only augmented in the LPS stimulation group but declined with only the transfection of siRNA-MYH9.

**FIGURE 4:**
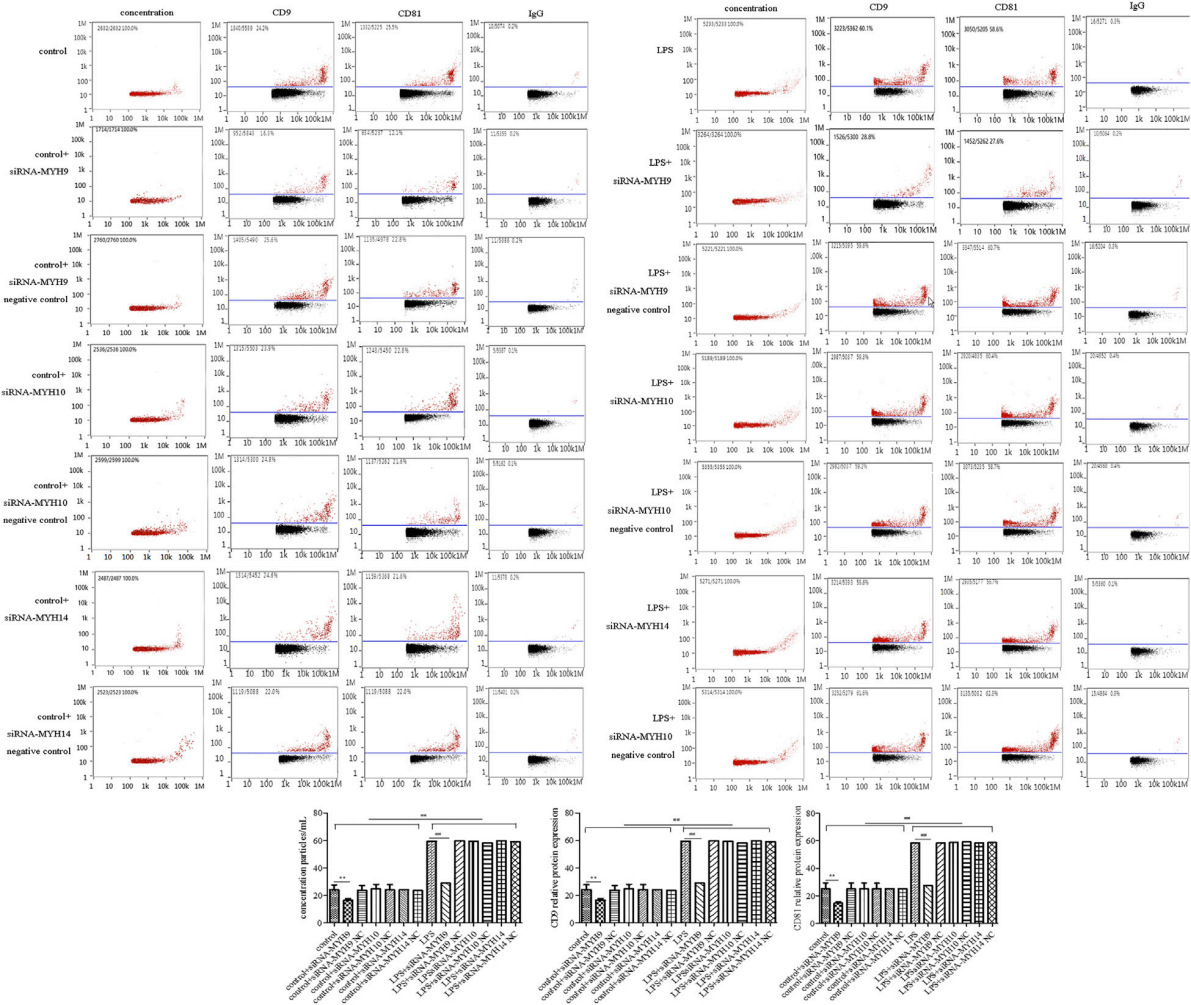
Transfection of siRNA-MYH9, but not siRNA-MYH10 or siRNA-MYH14 inhibited exosome release from microglial cells stimulated by LPS. For transfection, microglial cells were sub-cultured in 24-well plates at a density of 3 × 10^5^ cells/ml. The extracted exosomes were grouped based on transfection with siRNA or siRNA into microglial cells. Whether control or LPS stimulation cells were transfected with siRNA-MYH9, siRNA-MYH10, or siRNA-MYH14 at a final concentration of 100 nM for 48 h. Microglial cells stimulated by 1 mg/ml LPS for 24 h, microglial cells transfected with siRNA followed by 24 h LPS stimulation, microglial cells transfected with siRNA negative control followed by 24 h LPS stimulation. **(A)** Nanoparticle tracking analysis for the concentration of exosomes. **(B)** Flow cytometry analysis for CD9-labeled positive rate, CD81-labeled positive rate, or control FITC-IgG-labeled positive rate. Nine independent experiments were analyzed. A histogram depicted the quantitative representations of the protein expression levels of CD9 and CD81 for each group. The data were averages with SD, *n* = 9. ^**^ *p* < 0.01 vs. control microglial cells; ^##^ *p* < 0.01 vs. microglial cells stimulated with LPS.

Among the transfection group, only knockdown of NMMHC ⅡA in control or the LPS stimulation group (control + siRNA-MYH9: concentration: 6.60 ± 0.57 × 1,010 particles/ml, *p* < 0.01, CD9: 16.46 ± 1.21%, *p* < 0.01; CD81: 14.79 ± 0.89%, *p* < 0.01; LPS + siRNA-MYH9: concentration: 10.95 ± 0.62 × 1,010 particles/ml, *p* < 0.01, CD9: 28.99 ± 4.58%, *p* < 0.01; CD81: 27.50 ± 3.46%, *p* < 0.01) could decrease the elevated exosome concentration and the CD9 and CD81 protein labeled positive rates compared with that in control or the LPS stimulation group (control: concentration: 8.70 ± 0.48 × 1,010 particles/ml, CD9: 24.07 ± 3.69%; CD81: 25.14 ± 4.18%; LPS: concentration: 18.30 ± 1.06 × 1,010 particles/ml, CD9: 59.51 ± 3.66%; CD81: 58.51 ± 3.27%) (Figure 4), while knockdown of NMMHC ⅡB or NMMHC ⅡC did not change the above mentioned concentration and protein-labeled positive rates significantly compared to those of the LPS stimulation group (Figure 4).

**FIGURE 5:**
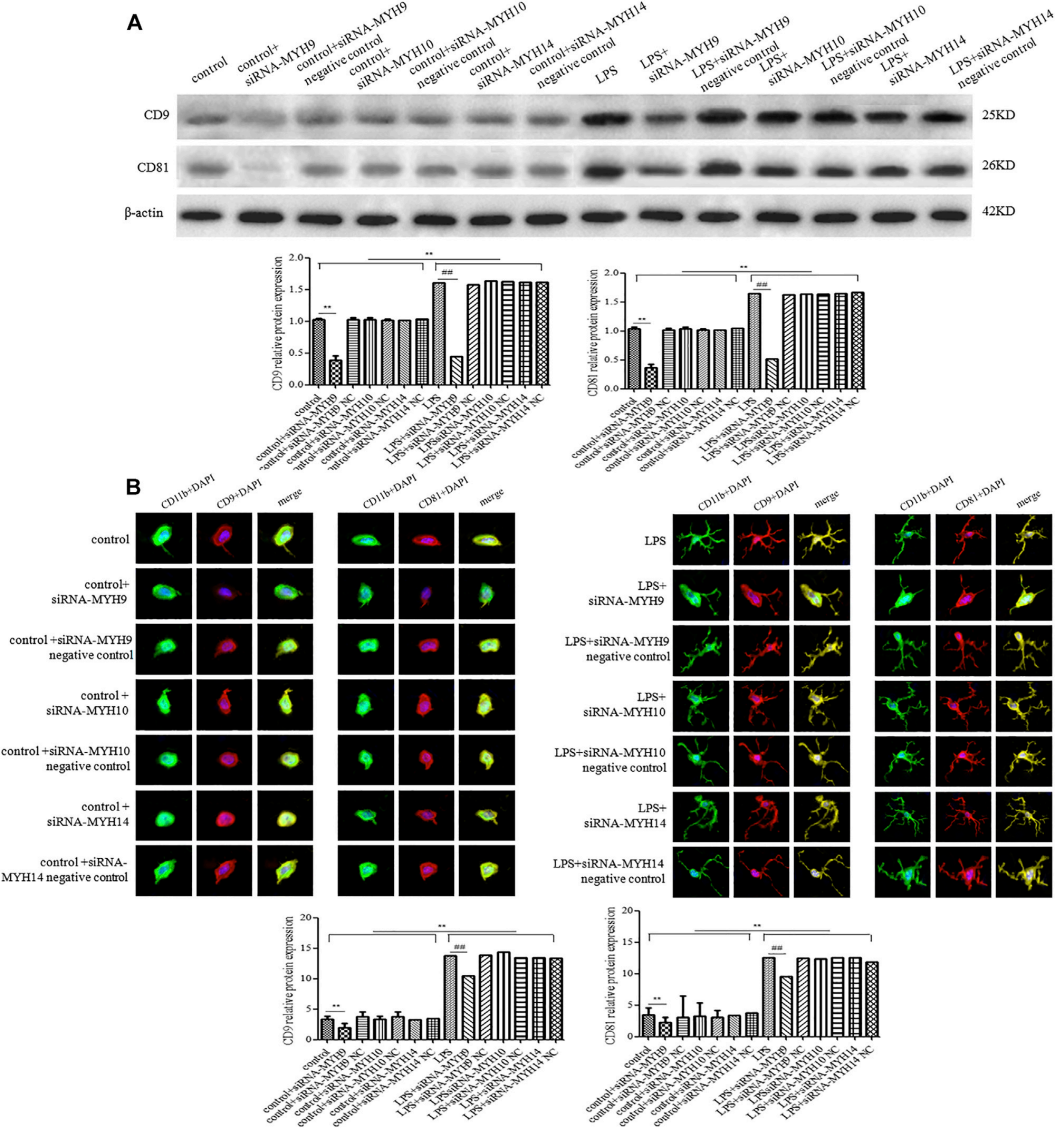
Transfection of siRNA-MYH9, but not siRNA-MYH10 or siRNA-MYH14 inhibited exosome release from microglial cells stimulated by LPS. For transfection, microglial cells were sub-cultured in 24-well plates at a density of 3 × 10^5^ cells/ml. The extracted exosomes were grouped based on transfection with siRNA or siRNA into microglial cells. Whether control or LPS stimulation cells were transfected with siRNA-MYH9, siRNA-MYH10, or siRNA-MYH14 at a final concentration of 100 nM for 48 h. Microglial cells stimulated by 1 mg/ml LPS for 24 h, microglial cells transfected with siRNA followed by 24 h LPS stimulation, microglial cells transfected with siRNA negative control followed by 24 h LPS stimulation. **(A)** The CD9 and CD81 protein expression levels for each group of exosome were examined by western blotting. Western blots were quantified. Nine independent experiments were analyzed. A histogram depicted the quantitative representations of the protein expression levels of CD9 and CD81 for each group. **(B)** Microglial cells were immunofluorescently stained with anti-CD9 or anti-CD81 (red), together with CD11b (green), and DAPI for nuclei (blue). Stained cells were examined by confocal microscopy. Scale bars = 10 μm. A histogram depicted the quantitative fluorescence intensities of CD9 or CD81 from a total of 45 randomly selected cells from nine independent experiments in each group of microglial cells. Nine independent experiments were analyzed. A histogram depicted the quantitative representations of the protein expression levels of CD9 and CD81 for each group. The data were averages with SD, *n* = 9. ^**^ *p* < 0.01 vs. control microglial cells; ^##^ *p* < 0.01 vs. microglial cells stimulated with LPS.

Meanwhile, the protein expression of CD9 and CD81 in control or the LPS group after transfection with siRNA-MYH9 were relative (control + siRNA-MYH9: CD9: 0.38 ± 0.07, *p* < 0.01, CD81: 0.36 ± 0.06, *p* < 0.01; LPS + siRNA-MYH9: CD9: 0.45 ± 0.03, *p* < 0.01, CD81: 0.51 ± 0.07, *p* < 0.01) compared with that in the control or LPS stimulation group (control: CD9: 1.02 ± 0.02, CD81: 1.04 ± 0.03; LPS: CD9: 1.60 ± 0.04, CD81: 1.64 ± 0.07), while only transfection of siRNA-MYH10 or siRNA-MYH10 could decrease the relative protein expression levels of CD9 and CD81 whether in control or the LPS stimulation group (Figure 5A). Microglial cells were immunofluorescently stained with anti-CD9 or anti-CD81 (red), together with CD11b (green), and DAPI for nuclei (blue) as observed under confocal microscopy. The fluorescence intensity of CD9 or CD81 from a total of 45 randomly selected cells from nine independent experiments in each group of microglial cells was quantified. Transfection of siRNA-MYH9 (control + siRNA-MYH9: CD9: 2.05 ± 0.75, *p* < 0.01, CD81: 2.14 ± 0.99, *p* < 0.01) could descend the fluorescence intensity of CD9 and CD81 in the control group (control: CD9: 3.38 ± 0.58, CD81: 3.34 ± 1.16) (Figure 5B). The fluorescence intensities of CD9 and CD81 were enhanced after the stimulation with LPS (LPS: CD9: 13.78 ± 1.04, *p* < 0.01, CD81: 12.47 ± 1.86, *p* < 0.01), while they declined only after treatment with siRNA-MYH9 (LPS + siRNA-MYH9: CD9: 10.38 ± 0.93, *p* < 0.01, CD81: 9.52 ± 0.82, *p* < 0.01) (Figure 5B).

### The NMMHC ⅡA-Regulated ROCK1/MLC/Actin Pathway Might Be the Potential Mechanism for Exosome Release

**FIGURE 6:**
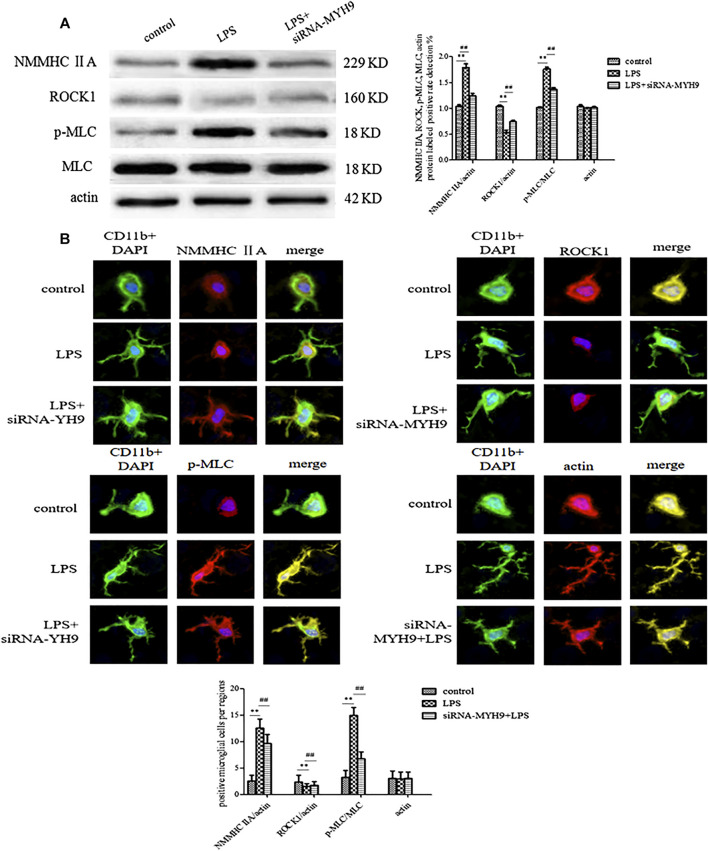
The NMMHC ⅡA-regulated ROCK1/MLC/actin pathway might be the potential mechanism for exosome release. The exosomes were divided on the basis of whether they were extracted from control microglial cells, microglial cells incubated with 1 mg/ml LPS for 24 h, and microglial cells transfected with siRNA-MYH9 at a final concentration of 100 nM for 48 h. **(A)** Microglial cells were immunofluorescently stained with anti-NMMHC ⅡA, anti-ROCK1, anti-MLC, or anti-actin, together with CD11b (green) and DAPI for nuclei (blue). Stained cells were examined by confocal microscopy. Scale bars = 10 μm. A histogram depicted the quantitative fluorescence intensity from five vision fields from nine independent experiments for each group of microglial cells. **(B)** The protein expression levels of anti-NMMHC ⅡA, anti-ROCK1, anti-MLC, and anti-actin were examined by western blotting. Western blots were quantified. A histogram depicted the quantitative representations of the protein expression levels of NMMHC ⅡA, ROCK1, MLC, and actin for each group. Nine independent experiments were analyzed. The data were averages with SD, *n* = 9. ^**^ *p* < 0.01 vs. control microglial cells; ^##^ *p* < 0.01 vs. microglial cells cultured for 24 h.

Western blotting and immunofluorescence experiments were performed to detect the change in expression levels of ROCK1/MLC/actin after transfection of siRNA-NMMHC ⅡA. The expression levels of NMMHC ⅡA and *p*-MLC/MLC were upregulated following the stimulation with LPS (NMMHC ⅡA: 1.79 ± 0.07, *p* < 0.01; *p*-MLC/MLC: 1.76 ± 0.04, *p* < 0.01), while ROCK1 (ROCK1: 0.53 ± 0.05, *p* < 0.01) was downregulated compared with the control group (NMMHC ⅡA: 1.24 ± 0.05; *p*-MLC/MLC: 1.37 ± 0.03; ROCK1: 0.74 ± 0.03). Actin, the skeleton of cells, was relatively stably expressed in each group (control group: 1.04 ± 0.03, LPS group: 1.01 ± 0.01; siRNA-MYH9 + LPS: 1.02 ± 0.02). The relative expression of NMMHC ⅡA stimulated by LPS accounted for nearly 150% of that in the control group. The transfection of siRNA-NMMHC ⅡA downregulated the abnormally elevated expression of NMMHC ⅡA and *p*-MLC/MLC (NMMHC ⅡA: 1.24 ± 0.05 *p* < 0.01; *p*-MLC/MLC: 1.37 ± 0.03, *p* < 0.01), and upregulated the relative expression of ROCK1 (ROCK1: 0.74 ± 0.03, *p* < 0.01) compared with the LPS stimulation groups (Figure 6A). Microglia in resting state demonstrated morphological changes by sensing changes in the environment as LPS. Microglia spread the branches and extended the longer branches with heterogeneous shapes upon stimulation with LPS. More heterogeneous shapes, such as spindle shapes, rod shapes, and branched shapes with CD11b fluorescently labeled microglia represented more amoeba-like forms. The fluorescence intensities of NMMHC ⅡA and MLC were enhanced by the stimulation with LPS (NMMHC ⅡA: 12.56 ± 1.67, *p* < 0.01; *p*-MLC/MLC: 15.00 ± 1.50, *p* < 0.01) compared with that in the control group (NMMHC ⅡA: 2.56 ± 1.13, *p*-MLC/MLC: 3.22 ± 1.30), meanwhile, the fluorescence intensity of ROCK1 (LPS: 1.56 ± 0.53, *p* < 0.01, control: 2.33 ± 1.32) declined, whereas, knockdown of NMMHC ⅡA had the opposite effect on fluorescent intensities (NMMHC ⅡA: 9.67 ± 1.73; *p*-MLC/MLC: 6.78 ± 1.30; ROCK1: 1.78 ± 0.67) (Figure 6B).

## Discussion

The data showed that the NMMHC ⅡA could interfere with exosome release from microglia stimulated by LPS. The quantitative measurement of exosome release was evaluated by changes in fluorescence intensity and relative protein expression. The administration of blebbistatin or transfection of siRNA-MYH9 could attenuate the increasing concentration and particle size of exosomes from microglia by stimulation with LPS, which could also decrease the CD9 and CD81-labeled fluorescence intensity and protein expression in exosomes. Microglia, the innate immune cells in the brain, were activated at the early stage of cerebral ischemia. Activated microglia rapidly responded to the stimulation to form an ameba shape, releasing a series of inflammatory factors into the CNS. Previous research found that 5 min after LPS stimulation, morphological changes in the microglia were observed, while 20 min after LPS stimulation, the cell bodies of neurons began to shrink. Thus, compared with neurons, microglia undergo apoptosis earlier in response to LPS stimulation (Lv et al., 2016). The microRNAs, miR-146b and miR-27a, contained in the micro-vesicles were found to be upregulated in the microglia stimulated by LPS. Conventional methods aimed at interfering with the expression levels of mRNA or proteins, such as direct protein inhibitors and siRNA or microRNA inhibitors that ultimately inhibit microRNAs, mRNAs, or proteins contained in the exosome were utilized. However, when one or more of the required attributes was interfered with, there existed other potential attributes which could trigger the same reactions. Exosomes are involved in cell-cell communication processes, so the measures to interfere with exosome release provide a new strategy for controlling diseases with exosome release as the origin. Currently, methods to reduce exosome release could comprehensively and effectively suppress the effectiveness of information contained in the exosome on the transmitted host cells or tissues.

Multivesicular bodies fuse with the plasma membrane, releasing intraluminal vesicles such as exosomes. Hence, the steps involved in their fusion with the membrane can be interfered with. The remodeling of cytoskeletal proteins such as microfilament bundles or proteins in focal contact fusion could contribute to membrane fusion. Previous literature indicated that GTPases, SNARE proteins, and calcium sensors contributed to membrane fusion into the cell. The heat shock protein, Hsp90 could mediate the fusion of multivesicular bodies with the plasma membrane via its exposed amphipathic helix (Lauwers et al., 2018). KIBRA could control exosome secretion via the inhibition of the proteasomal degradation of Rab27a (Song et al., 2019). Exosome release was also negatively regulated by mTORC1 in response to changes in growth factor conditions (Zou et al., 2018). RalA and RalB GTPases were considered as novel regulators of multivesicular body formation and exosome release (Hyenne et al., 2015). HOTAIR could facilitate the final step of fusion by influencing VAMP3 and SNAP23 colocalization via rapamycin (mTOR) signaling (Yang et al., 2019). TSG101 promoted the aggregation and degradation of multivesicular body proteins to restore exosome secretion (Villarroya-Beltri et al., 2016). Further analyses that needed additional research were employed in the study of exosome release mechanism of cells. Meanwhile, qualitative analysis was mainly employed in the study of driving mechanism, which needed more accountability analysis and model analysis.

Myosin, a kind of ATP-dependent molecular motor in eukaryotic cells, plays an important role in cell movement and intracellular material transport. Actin-associated proteins have roles in the formation of exosomes and the regulation of signaling functions of the exosome in osteoclasts (Holliday et al., 2019). When the filament slides, the head of myosin contacts the molecules of actin, and provides the required mechanical forces for cytoplasmic flow, organelle movement, material transport, and mitosis, all of which participate in cell phagocytosis and movement. The addition of blebbistatin or transfection of MYH9 could attenuate the increasing exosome release by the stimulation with LPS. On the determination of subtypes, only MYH9, not MYH10 or MYH14 could regulate the release of exosomes. The exosome concentration and positive rate of labeled proteins demonstrated that LPS could increase the amount of released exosomes, while knockdown of NMMHC ⅡA could increase the exosome concentration and particle size. Quantification of fluorescence intensity and protein expression of CD9 and CD81 again verified that MYH9 might be the subtype of myosin that participates in exosome release. Nonmuscle myosin ⅡA binds to actin to form the molecular motor of a cell. The heavy chain of NMMHC ⅡA provides ATPase activity and the binding site of actin and the light chain (Pecci et al., 2018). NMMHC Ⅱ A specifically exists in platelets, lymphatic cells, and neutrophils; it is encoded by the MYH9 gene. It has recently been demonstrated that NMMHC Ⅱ A plays a critical role in cell migration, adhesion, immune response of the body, tumors occurrence and development, and other pathological processes. NMMHC Ⅱ A is the key mechanical connection between lymphocyte function-associated antigen 1 and cytoskeleton, which promotes the adhesion of LFA-1 in lymphoid tissue (Morin et al., 2008). NMMHC Ⅱ also affected cellular rolling with abdominal foot delayed response of antigen recognition (Jacobelli et al., 2004). The role of NMMHC Ⅱ A in the response to the immune system cannot be ignored; it is largely expressed in lymphocytes, neutrophils, and monocytes. The interaction between NMMHC ⅡA and immune cells is involved in neuroinflammation. Microglia belong to the mononuclear phagocyte system; through the secretion of their exosomes, they are involved in intensifying the inflammatory response. NMMHC ⅡA might be a promising candidate for interfering with exosome release from microglial cells upon stimulation by LPS.

Rho-associated kinase 1 (ROCK1) is a key regulator of actin cytoskeleton and cell polarity. ROCK1 also directly induces the phosphorylation of myosin light chains (MLC) or inactivates the phosphorylated myosin phosphatase-binding subunit (MYPT), thereby directly or indirectly increases MLC phosphorylation (Coleman et al., 2001; Sebbagh et al., 2001). Moreover, MLC phosphorylation induces the cleavage of a Rho GTPase effector and ROCK1, which induces a constitutive kinase activity by removing the inhibitory domain (Chang et al., 2006). Previous studies demonstrated that the NMMHC ⅡA-actin interaction regulated the caspase-3/ROCK1/MLC signal cascade by a feedback mechanism upon oxidative stress-induced neuronal apoptosis (Shen et al., 2015; Wang et al., 2017). Our present findings confirmed that NMMHC ⅡA might trigger the ROCK1/MLC/actin signaling pathway to suppress exosome release. Proteomics showed that exosomes shed by osteoclasts are rich in actin and actin-associated proteins, such as myosin heavy chain, myosin, actinin, and capping protein, etc. (Holliday et al., 2019) Actin-myosin contraction might play a role in the scission of the exosome (Rody Jr et al., 2019; Wang et al., 2019). Actin binds either ATP, ADP-Pi, or ADP. ATP-actin polymerizes and facilitates the formation of filaments in cells by causing a change in conformation (Holliday et al., 2019). A proteomic study showed that NMMHC ⅡA is a common myosin to be found in exosomes; it was identified as a potential exosome biomarker for inflamed trigeminal satellite glial cells (Vinterhoj et al., 2019). The abundant NMMHC ⅡA could be implicated in exosome formation; myosin-actin contraction might be required for exosome formation and movement.

The ROCK1/MLC/actin pathway regulated by NMMHC ⅡA might be a potential mechanism for exosome release. The function of exosomes can be regulated by many interfering processes, such as the destruction of fusion with the cellular membranes, damaging the releasing exosomes, or blocking exosome release, etc. The mechanism by which NMMHC ⅡA conducts exosomes could be elucidated in further research. In the present study, NMMHC ⅡA could attenuate the elevated exosome release from microglial cells stimulated by LPS. NMMHC ⅡA might affect the release of exosomes by triggering the ROCK1/MLC/actin signaling pathway.

## Data Availability Statement

The datasets presented in this article are not readily available because requests to access the datasets should be directed to yannilv225@ncu.edu.

## Ethics Statement

The animal study was reviewed and approved by The First Affiliated Hospital of Nanchang University.

## Author Contributions

All authors contributed toward data collection, data collation, drafting, editing, and statistic analysis.

## Funding

This work was supported by the Foundation Project: National Natural Science Foundation of China (No.: 81760094, 31860276), China; the Foundation of Jiangxi Provincial Department of Science and Technology Applied research cultivation projects (No. 20181BBG78051, No. 20171BAB215067); the Foundation of Jiangxi Provincial Department of Science and Technology Youth Key Project (No. 20202ACBL206001); the Project of Jiangxi health and Family Planning Commission (No.: 20203114, 20203095, 20181021), China; the scientific research project of traditional Chinese medicine in Jiangxi Province (No.: 2018B130); and the Open Project of Key Laboratory of Modern preparation of TCM, Ministry of Education Jiangxi University of Traditional Chinese Medicine (TCM-2019010).

## Conflict of Interest

The authors declare that the research was conducted in the absence of any commercial or financial relationships that could be construed as a potential conflict of interest.
