# Corrigendum: Nonmuscle myosin heavy chain IIA-mediated exosome release via regulation of the rho-associated kinase 1/myosin light chains/actin pathway

**DOI:** 10.3389/fphar.2024.1465932

**Published:** 2024-09-20

**Authors:** Yanni Lv, Jin Chen, Jinfang Hu, Yisong Qian, Ying Kong, Longsheng Fu

**Affiliations:** ^1^ Department of Pharmacy, The First Affiliated Hospital of Nanchang University, Jiangxi, China; ^2^ Department of Neurology, The First Affiliated Hospital of Nanchang University, Jiangxi, China; ^3^ Institute of Translational Medicine, Nanchang University, Nanchang, China

**Keywords:** nonmuscle myosin heavy chain IIA, exosome release, rho-associated kinase 1/myosin light chains/actin, microglial cells, lipopolysaccharide

In the published article, there was an error in Figure 2 as published. In Figure 2B, the panel representing CD18 in the LPS+blebbisatin group was a repeat of the panel representing CD9 in the LPS+blebbisatin group. The corrected Figure 2 and its caption appear below.

**FIGURE 2:**
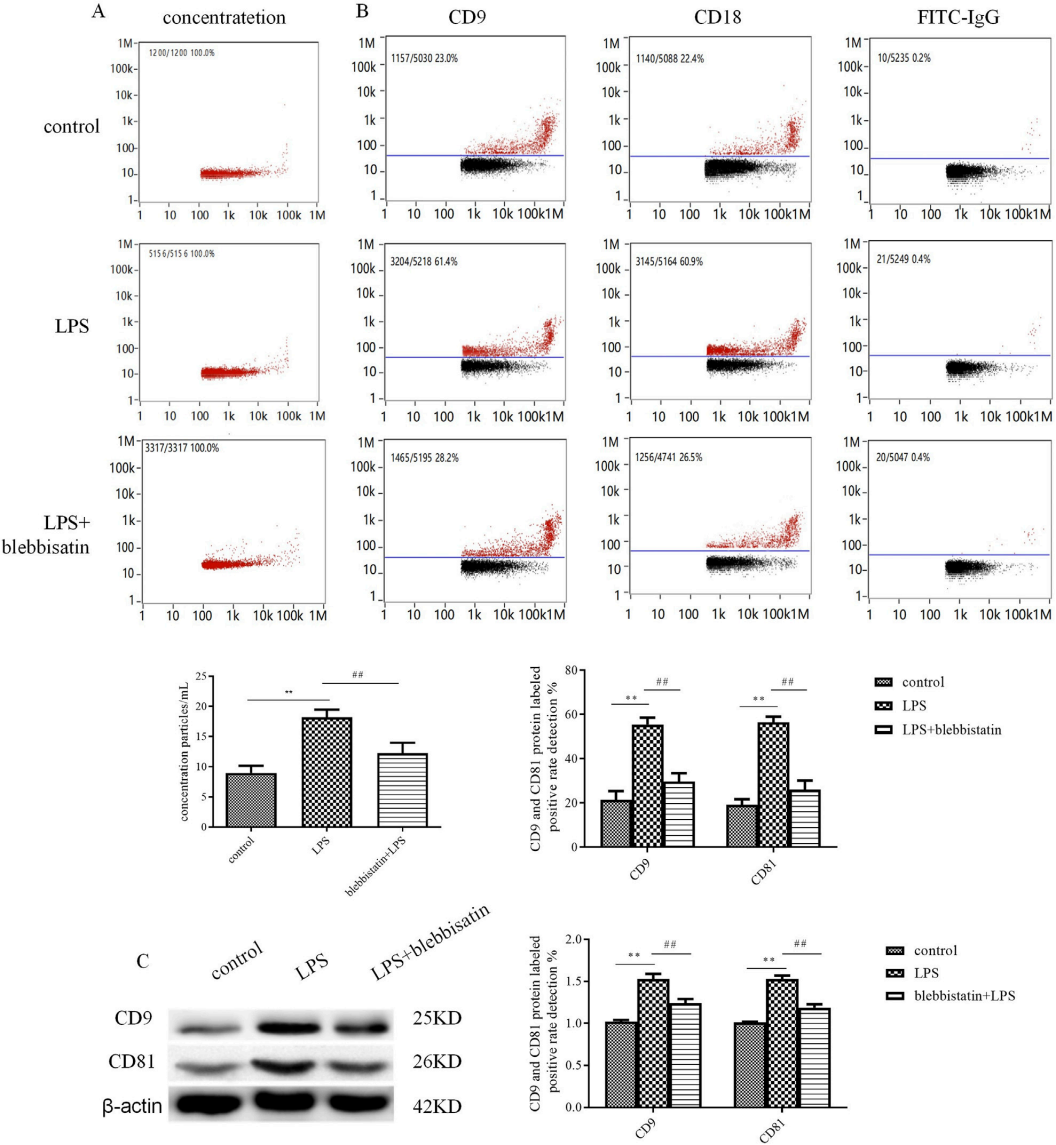
The myosin inhibitor, blebbistatin, inhibited exosome release from microglial cells under the stimulation of LPS. The extracted exosomes were divided into those extracted from control microglial cells, microglial cells incubated with 1 mg/mL LPS for 24 h, and microglial cells added with 1 μM blebbistatin 0.5 h before the 24 h stimulation of LPS. **(A)** Nanoparticle tracking analysis for the concentration of exosomes. **(B)** Flow cytometry analysis for CD9-labeled positive rate, CD81-labeled positive rate, or control FITC-IgG-labeled positive rate. **(C)** The protein expression levels of CD9 and CD81 exosome marker proteins were examined by Western blot. Western blots were quantified. Nine independent experiments were analyzed. A histogram depicts the quantitative representation of CD9 and CD81 protein expression for each group. The data were averages ±SD, n = 9. **p < 0.01 vs. control microglial cells; ^##^ *p* < 0.01 vs. microglial cells stimulated with LPS.

The authors apologize for this error and state that this does not change the scientific conclusions of the article in any way. The original article has been updated.

